# Displacement of Tendons

**Published:** 1902-07-19

**Authors:** 


### DISPLACEMENT OF TENDONS.

In a recent clinical lecture Mr. Howard Marsh [1] drew attention to an injury of which but little mention is made in the ordinary surgical text-books, namely, the accidental displacement of tendons. First of all, he says, it must be understood that this is one of the rarest accidents in minor surgery, so that one must not be too ready to accept it as a cause of the symptoms in any given case unless one can be pretty sure that they cannot be explained in some other way. The clearest instance of the slipping of a tendon is that of the peroneus longus. When the foot is turned in, the tendon cannot slip out of place, but if in jumping or dancing the foot comes to the ground with some force while in a turned-out position, the tendon is pulled out to the edge of the external malleolus, and is there held only by a ligamentous band; and if this band should be torn through, the tendon slips on to the front of the malleolus. The treatment of this accident is to replace the tendon, put the foot in a position of inversion, and encase it in a light plaster of Paris or leather splint for a month, and use massage and passive movement. The tibialis posticus is another tendon which sometimes, but very rarely, becomes displaced, and the same may be said of the popliteus. Then there is the long tendon of the biceps. If in the act, for instance, of wringing clothes the humerus be forcibly rotated outwards by the biceps acting as a supinator, the tendon may slip over the inner tuberosity of the humerus, and if not replaced it may acquire a new attachment to the bone. A patient again may complain of something slipping over his knuckle. This is the extensor communis digitorum slipping to one side or the other as the finger is flexed and extended. In a recent case the finger should be kept extended on a splint for three weeks. Other curious cases of slipped muscles and tendons are mentioned. A girl was looking out of a second-floor window when she suddenly twisted her head in order to look up at her brother at a third-floor window. Her head became suddenly fixed in that position. A boy was washing, and on twisting his head it suddenly became fixed, and he was unable to move it. A man was riding in a race and was leading. He turned his head for a moment to see how near his opponent was, when his head became fixed, and he finished the race looking back over his shoulder. In such cases what happened was that one of the small tendons which are inserted into the prominent tubercles of the transverse processes of the cervical vertebræ slips out of place. The treatment consists in putting the patient under an anæsthetic, kneading the deep muscles in the side of the neck, and moving the head freely in all directions.

[1] St. Bartholomew's Hospital Journal.

